# Clinical Status and Future Prospects of Transanal Total Mesorectal Excision

**DOI:** 10.3389/fonc.2021.752737

**Published:** 2022-01-03

**Authors:** Zichao Guo, Xiaopin Ji, Shaodong Wang, Yaqi Zhang, Kun Liu, Changgang Wang, Yang Deng, Tao Zhang, Xi Cheng, Ren Zhao

**Affiliations:** ^1^ Department of General Surgery, Ruijin Hospital, Shanghai Jiao Tong University School of Medicine, Shanghai, China; ^2^ Shanghai Institute of Digestive Surgery, Ruijin Hospital, Shanghai Jiao Tong University School of Medicine, Shanghai, China

**Keywords:** low rectal cancer, transanal total mesorectal excision (taTME), laparoscopic rectal surgery, minimally invasive surgery (MIS), colorectal cancer

## Abstract

Low rectal cancer has always posed surgical challenges to gastrointestinal surgeons. Transanal total mesorectal excision (taTME) is a novel approach to radical resection for low rectal cancer. Compared with conventional laparoscopic TME (laTME), taTME is relevant to the benefits of better vision of the mesorectal plane, feasibility of operating in a narrow pelvis, and exact definition of distal resection margin, which may lead to a higher possibility of free circumferential resection margin, better quality of TME specimen, and lower conversion rate. Although there are concerns about its long-term oncological outcomes and complex learning curve, taTME is a promising alternative for rectal cancer. In this review, we discuss the application status and prospects of taTME.

## Introduction

Rectal cancer is one of the most common cancers worldwide. The rectum is anatomically enfolded in a fatty tissue coverage known as the mesorectum, which lies in the pelvis following the sacrum and shapes to the anal canal (1). Colorectal cancer is one of the leading causes of mortality in America, with the incidence projected to continue to increase (2). The narrow pelvic space, which hinders ideal tumor resection, has always posed surgical challenges to the gastrointestinal surgeon. It is crucial to perform surgery through the correct mesorectal plane when treating rectal cancer (3). Since its inception and validation, total mesorectal excision (TME) and neoadjuvant chemoradiotherapy have become the standard treatments for CRC (4). In the early 1990s, laparoscopic surgery has become prevalent and has been validated in CRC, with benefits of faster recovery, better cosmetic effect, less postoperative pain, shorter hospital stay, and fewer complications (5–7).

After the first TME surgery was advocated by Bill Heald (1) in 1982, the principle of TME, the development of medical science and minimally invasive surgery (MIS), the experience of transanal endoscopic microsurgery (TEM) and transanal MIS (TAMIS), the method of transabdominal and transanal (TATA) (8, 9), and the concept of natural orifice transluminal endoscopic surgery (NOTES) inspire surgeons to explore a new operation, which is taTME. By its unique transanal approach for dissection, taTME is relevant to the benefits of accurate exposure of the mesorectal plane, direct vision of distal resection margin (DRM), and feasibility of overcoming technical difficulties in the narrow pelvis; thus, it may promise a higher possibility of free circumferential resection margin (CRM) and DRM, a better quality of TME specimen, and better functional outcomes over conventional TME, especially when treating male patients, obesity, narrow pelvis, and large tumors. In addition, the short-term prognosis after taTME is not inferior to that of the conventional TME (10, 11).

In this review, we aim to discuss the evolution, research status, controversy, and prospects of taTME.

## The Evolution of taTME

### The History and Consensus of taTME

In 1982, the first TME was advocated by Bill Heald, which was labeled as a milestone in the history of rectal cancer surgery (1). In 2007, Whiteford et al. (3) first successfully performed TEM for rectal resection on newly thawed cadavers, which was a surgical technique of transanal local resection for early rectal cancer, using an endoscopic technique to avoid transabdominal major resections, stoma creation, and potential complications in patients with pT1N0 rectal cancer after accurate diagnosis and staging. TEM has become a hot topic in the field of surgical treatment for rectal cancer (12). TAMIS is a method of inserting a single-incision laparoscopic port into the anus and using conventional laparoscopic instruments for operation (13). The specimens resected by TEM or TAMIS can be dragged out directly through the anus, rather than through another abdominal incision, thus preventing surgical complications such as incision infection, hernia, and tumor cell implantation (13), which coincides with the concept of NOTES. The development of TATA (8, 9) makes low rectal resection and anastomosis possible. However, this method only solves the problem of anastomosis. The exposure of the low rectum and the quality of specimens, especially the quality of DRM, have not been improved (14). Advances in medical science have facilitated the development of the MIS. The principle of TME, the development of MIS, TEM, and TAMIS, the limitations of TATA, and the concept of NOTES inspired surgeons to explore a new operation, namely, taTME. In 2013, on the basis of laparoscopic-assisted taTME (LA-taTME), Dr. Zhang Hao et al. (15) from China and Leroy et al. (16) from France reported two cases of rectal resection using a complete transanal approach one after another. In the same year, Professor Heald (17) published a review called “A new solution to same old problems: transanal TME”, affirming the prospects of taTME.

There is no internationally recognized definition of taTME. It is suggested that taTME should be defined as a bottom-up transanal rectal resection surgery using a TEM or TAMIS platform, following the principle of TME (18). It is recommended to abbreviate this surgery to taTME, in which “TME” is basic operation and “ta” is the modifier word to describe the transanal approach. Without special instructions, taTME usually refers to LA-taTME. When assisted by a robotic system instead of laparoscopy, the operation is called robot-assisted taTME (RA-taTME). NOTES-taTME was used to describe the operation using a complete transanal approach.

The proclaimed standard procedures for LA-taTME normally begin with transabdominal laparoscopic dissection. It can also be operated transanally, first or simultaneously, from above and below with two surgical teams.

**Figure 1:**
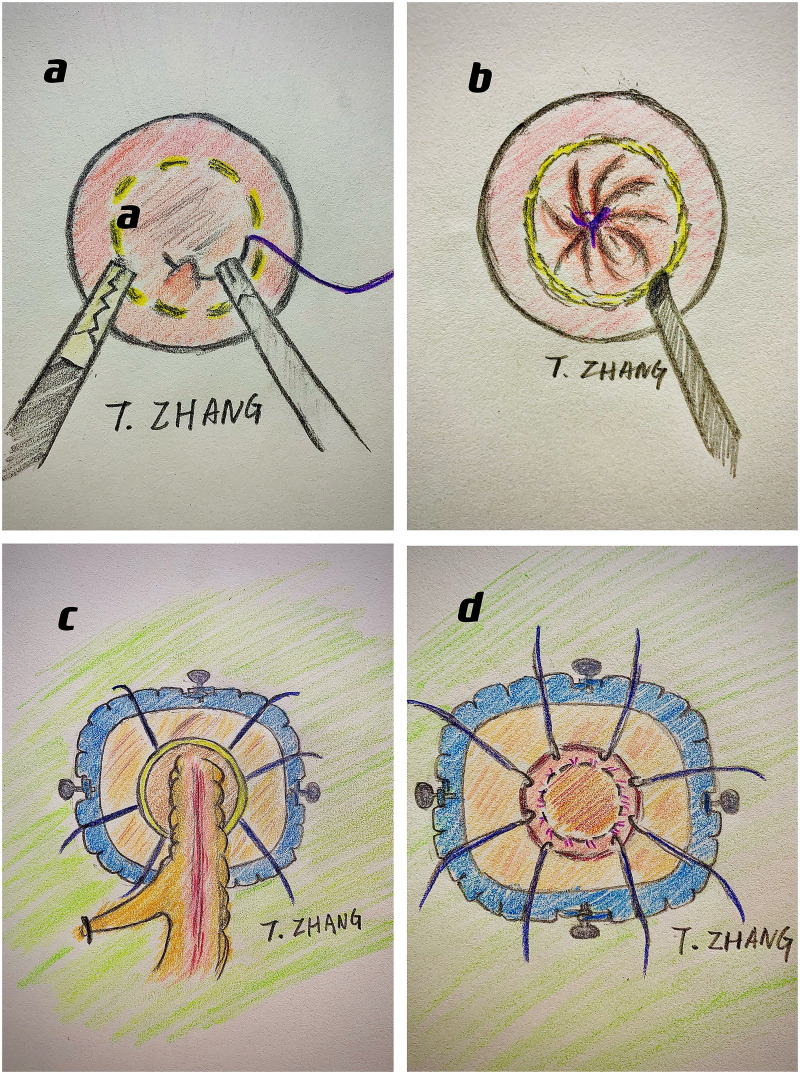
The surgical procedures of taTME: **(A)** do purse-string suture at pre-marked distal margin; **(B)** dissect along the planned cutting line; **(C)** meet with the abdominal anatomy and drag out the specimen through anus; **(D)** anastomosis with either stapler or stitches.

With a customized metal sleeve for rectoscopy and corresponding equipment, the significant advantage of the TEM platform is its stability (3, 12). However, it restricts the transformation of surgical fields and the utilization of conventional laparoscopic instruments. The TAMIS platform is more prevalent in taTME based on single-incision laparoscopic surgery (19, 20). It does not require customized equipment and can utilize the single-incision laparoscopic surgery port and conventional instruments for operation (**Figure 1**).

### The Learning Curve, Indications, and Contradictions of taTME

In terms of the complex learning curve of taTME, it is recommended by consensus and guidelines (18, 21–24) that this operation should be performed by certified surgeons with adequate experience of colorectal laparoscopic surgeries in large-volume colorectal centers. The experience of managing 30 to 60 cases has been reported to be adequate for introduction. Surgeons can achieve stable outcomes after a minimum of 50 cases are performed as primary operators. During this period, structured training programs and refined taTME protocols were essential (25).

After comprehensive considerations, indications and contradictions of taTME have been established and are constantly being refined by experts worldwide (18, 22, 23). For rectal cancer, taTME can be considered as a priority when dealing with male patients, obesity, narrow or deep pelvis, prostatic or mesorectum hypertrophy, low and anterior tumor, and tumor size >4 cm. For benign rectal diseases, taTME may be a better option when dealing with large tumors, inflammatory bowel diseases, radiation proctitis, familial adenomatous polyposis, rectal strictures, or complex fistulae.

Contradictions include obstructive rectal tumors, T4 tumors, and a history of anal strictures or injuries. In addition, taTME is not recommended when the tumor is above the peritoneal reflection.

## The Research Status of taTME

### The Short-Term Outcomes of taTME

As an innovative surgical approach, the short-term prognosis of taTME has attracted attention. Roodbeen et al. (26) found that after accurate staging, case recruitment, and discharge, the 2-year local recurrence (LR) rate after taTME was 3% (95% CI =2–5), which was acceptable. Yao et al. (27) included 1,283 taTME cases registered in the Chinese taTME Registry Collaborative (CTRC) from May 2010 to November 2019 for analysis. The results showed that 81.9% of specimens were complete and the rate of positive CRM was 2.8%, while the abdominal and perineal conversion rates were 0.5% and 1.9%, respectively. The 2018 CTRC annual report (19), which conducted retrospective and prospective analyses of 601 taTME cases, indicated that taTME was associated with an integral specimen and the probability of free CRM and DRM. The reports conducted by Lacy et al. (28) and Penna et al. (29), based on the International taTME Registry, including 186 and 720 cases, respectively, showed similarly acceptable short-term outcomes and good specimen quality after taTME. These findings indicated that the oncological short-term outcomes after taTME were acceptable and that taTME may be a promising alternative for rectal cancer.

The comparison between taTME and laTME has also raised concerns. Detering et al. (30), based on Dutch ColoRectal Audit, found that the rates of positive CRM were similar between the taTME and laTME groups (4.3% vs. 4.0%, *p* = 1.000), and the conversion rate in the taTME group was significantly lower than that in the laTME group (1.5% vs. 8.6%, *p* < 0.001). A meta-analysis by Lin et al. (31), including 899 cases from 12 retrospective case–control studies, found no significant difference in oncological outcomes between the taTME and laTME groups, including positive CRM, positive DRM, quality of specimen, temporary stoma, or LR. Similar results were reported by Rubinkiewicz et al. (32). In addition, the study by Zeng et al. (33), based on a randomized controlled trial (RCT), showed that positive DRM was detected in two cases in the laTME group (1.5%), while none was reported in the taTME group (*p* = 0.498), and the length between the tumor and DRM in the taTME group (1.4 ± 1.1) may tend to be longer than that in the laTME group (1.3 ± 0.9, *p* = 0.745). These findings indicate that compared with well-established laTME (5–7, 14), taTME yields non-inferior oncological short-term outcomes, considering its implementation phase. Although there is still a lack of literature, further exploration may validate the superiority of taTME in oncological regional control and long-term outcomes.

The comparison between taTME and robotic-TME is another concern. Lee et al. (34) conducted a case-matched comparison of 730 rectal cancer patients who received taTME or robotic-TME in five high-volume referral centers from 2011 to 2017. The results showed that there was no significant difference in the quality of TME specimens and the rates of positive CRM (5.6% vs. 6.0%, *p* = 0.839). However, the rate of positive DRM may be higher after taTME (1.8% vs. 0.3%, *p* = 0.051). This could be related to the steep learning curve of taTME and more caution should be paid to the exact determination of DRM, although the difference was not statistically significant. Compared with robotic-TME, the current literature suggests that taTME has non-inferior oncological short-term outcomes.

### The Preoperative Assessment and Postoperative Complications of taTME

The 2015 (35) and 2019 Chinese consensus (18), as well as the Canadian taTME expert collaborative statement (23), have detailed descriptions of the indications and contraindications of taTME, but new findings may provide a new dimension. Roodbeen et al. (36) conducted an analysis of 2,653 taTME cases based on the International taTME Registry from July 2014 to January 2018, among which there were 107 cases of positive CRM (4.0%). Univariate and multivariate analyses showed that there were five factors closely related to positive CRM after taTME: tumors within 1 cm from the anus, anterior tumors, cT4 tumors, extramural venous invasion (EMVI), and involved CRM reported by preoperative baseline MRI. Another multivariate analysis by Penna (37) showed that the independent risk factors of anastomotic failure were male sex, obesity, smoking, diabetes mellitus, tumors >25 mm, excessive intraoperative blood loss, manual anastomosis, and prolonged perineal operation time. In addition, the 2018 CTRC also conducted an analysis of the risk factors of postoperative complications after LA-taTME (38). A total of 857 patients were recruited, and 563 cases were included and analyzed. Univariate and multivariate analyses showed that the independent risk factors of anastomotic leakage after LA-taTME were anastomosis without a stapler (*p* = 0.004), not creating a prophylactic stoma (*p* = 0.009), and probably tight spleen flexure (*p* = 0.103). These findings may be of significance for preoperative assessment and perioperative clinical decision-making of taTME and of help to reduce the incidence of positive CRM and anastomotic complications (**Tables 1**–**3**).

**Table 1:** Multivariate analysis of postoperative anastomotic leakage of 563 cases after LA-taTME.

Variants	Regression Coefficient	OR (95% CI)	*p*-value
Anastomosis by stapler	−1.08	0.340 (0.163–0.708)	0.004
Prophylactic stoma	−0.932	0.394 (0.195–0.794)	0.009
Loose spleen flexure	−1.016	0.362 (0.107–1.228)	0.103

**Table 2:** Postoperative complications of 563 cases after LA-taTME.

Complications	Number of Cases (%)
Total Postoperative Complications	115 (20.4%)
Anastomosis leakage	43 (7.6%)
Level A (do not need specific treatment)	11 (2.0%)
Level B (need non-surgical treatment)	14 (2.5%)
Level C (need surgical intervention)	14 (2.5%)
Not graded	4 (0.7%)
Postoperative bowel obstruction	14 (2.5%)
Uroschesis	8 (1.4%)
Postoperative bleeding	7 (1.2%)

**Table 3:** Univariate analysis of postoperative anastomotic leakage of 563 cases after LA-taTME.

Variants	Number of Cases	Number of Cases of Anastomosis Leakage (Total 43 cases)	*x*² value	*p*-value
Anastomosis by stapler			3.128	0.077
Yes	440	29 (6.6%)		
No	123	14 (11.4%)		
Prophylactic stoma*			7.139	0.008
Yes	309	16 (5.2%)		
No	237	27 (11.4%)		
Loose spleen flexure*			3.232	0.072
Yes	97	3 (3.1%)		
No	454	38 (8.4%)		

*Partial data is missing.

The main postoperative complication of rectal cancer is anastomosis failure. Detering et al. (30) found that the difference in anastomotic leakage rate between the taTME and laTME groups was not statistically significant (16.5% vs. 12.2%, *p* = 0.116). A meta-analysis by Lin (31) or Rubinkiewicz (32) also showed that the overall intraoperative and postoperative complications after laTME or taTME were similar, and there was no significant difference in blood loss, conversion rate, operative time, anastomotic leakage, bowel obstruction, or urinary morbidity. Using the transanal approach, the exact definition of resection margin, protecting ureter, nerve vascular bundles, and pelvic plexus, and preserving sphincters are easier to achieve under direct vision and accurate exposure. Therefore, they may promise non-inferior outcomes of complications.

### The Application Status of Robotic-Assisted taTME

Robotic-assisted colorectal surgery has caused a considerable upsurge since the successful introduction of robotic systems in surgical fields. The advantages of robotic systems, including 3D vision, flexible movement, and reduction of tremor transmission, promise a better instrument maneuverability and stability in a narrow surgical field for fine anatomy (39). A robotic system is usually utilized for the transabdominal part in the implementation phase of the RA-taTME (40, 41). It may help to pass the steep learning curve and cut down the expenditures and personnel costs while simultaneously operating transabdominally and transanally, which require two surgical teams. A robotic system that was utilized for the transanal part has also been reported (40, 42–44), replacing the TEM or TAMIS platform. The advantages of robotic systems are reported to be helpful in overcoming the technical difficulties of low rectal resection and anastomosis, thus achieving good regional control.

Although there have been few small-scale studies on the application of robotic systems in taTME, the results are inspiring, in terms of the quality of TME specimens, the number of harvested lymph nodes, and the conversion rate (40–44). Further exploration of the RA-taTME and customized robotic systems is expected.

### The Controversy of taTME

Much attention has been paid to the short-term prognosis and oncological and pathological outcomes of taTME. However, there are few reports from large-volume rectal cancer centers that focus on mid- and long-term prognosis and oncological outcomes of taTME. There is still a lack of high-level data from RCTs to support taTME. Roodbeen et al. (26) conducted a multicenter cohort study in six tertiary referral centers. The results showed that among 767 cases eligible for analysis, 24 cases had local recurrence after a median follow-up of 25.5 months, with an actuarial cumulative 2-year LR rate of 3% (95% CI = 2–5). An acceptable oncological regional control after taTME shows non-inferiority compared with the conventional TME. However, the opposite outcome of the first nationwide study from Norway raised the main controversy regarding taTME. Wasmuth et al. (45) reported 12 cases of LR (7.6%) in a total of 157 cases after taTME was performed in Norway from October 2014 to October 2018, eight of which manifested as multifocal or extensive growth. The LR rate after taTME was significantly higher than that after conventional TME (3.4%), with a short recurrence time (average, 11 months). The recurrence is characterized by rapid and multifocal growth in the pelvic cavity and lateral wall, which is different from typical manifestations. This may be related to the steep learning curve of taTME and differences in patients’ general status between the taTME group and routine surgery group, such as sex, BMI, tumor size, and proportion of patients receiving neoadjuvant chemoradiotherapy. Norwegian health authorities announced a moratorium on the application of taTME. Nonetheless, another study from the Netherlands may be explanatory and enlightening. Oostendorp et al. (46) included the first 10 taTME cases in 12 centers during their implementation phase. After a median follow-up of 21.9 months, the overall LR rate was 10%, with a mean (S.D.) recurrence time of 15.2 months. Among them, eight presented with multifocal growth. However, the overall LR rate decreased to 5.6% in the prolonged cohort and continued to decline to 4.0% after excluding the first 10 cases from each center. These findings indicate that the learning curve of taTME may be more complicated than expected. A larger sample size, a longer follow-up period, and centralization of this technique are suggested for validation in further exploration. Particular emphasis should be placed on quality control, surveillance, and refined taTME protocols. Previous experiences of MIS and transanal surgery and formatted instructions for taTME are critical factors that make a difference (25, 47). Although there is currently a lack of high-level evidence, the latest meta-analysis (48, 49) still stands for the noninferiority of taTME.

Argument also increased the quality of life and functional outcomes of patients after taTME. A meta-analysis by Heijden et al. (50) showed that there was no significant difference in the probability of patients undergoing low anterior resection syndrome (LARS) after laTME or taTME (*p* = 0.18). Koedam et al. (51) conducted a prospective analysis of quality of life and functional outcomes after taTME. It showed a similarity at the 6-month postoperative point compared to the preoperative baseline, except that social function and anal pain remained significantly worse. Another analysis by Veltcamp Helbach et al. (52) comparing functional outcomes between the taTME group and laTME group showed that LARS scores seemed to be higher in the taTME group at 6 months post-stoma closure, although not statistically significant. In addition, these two groups presented similar outcomes in other fields, such as sexual function and urination. It should be pointed out that cases in the taTME group are more likely to experience a low anastomosis, which may result in higher postoperative LARS scores.

## The Prospects of taTME

Using a unique transanal approach for dissection, taTME is relevant to the benefits of direct vision of DRM, accurate exposure of the mesorectal plane, and wider operating space in the narrow pelvis, which contributes to the exact definition of the resection margin, protecting the ureter, nerve vascular bundles, and pelvis plexus, and preserving sphincters; thus, it may promise a higher possibility of free CRM and DRM, a better quality of TME specimen, and better functional outcomes than conventional TME. After taTME, the specimen can be dragged out though the anus, which coincides with the concept of NOTES and the trend in surgical techniques from MIS to non-invasive treatment. Although there is a lack of high-level evidence, current large-scale studies have indicated non-inferiorities in the oncological and functional outcomes of taTME. Generally, taTME is a novel, feasible, and promising alternative surgical approach for rectal cancer that is still under investigation.

However, there are still some difficulties with taTME. First, NOTES-taTME cannot be used to explore the abdomen and ligate the root of blood supply vessels before transanal resection. Second, the learning curve of taTME is longer and more complex, especially that of NOTES-taTME. Lastly, the systematic and formatted training programs (21, 25), standardized guidelines, and refined protocols of taTME, as well as customized instruments and surgical platforms are expected to be improved.

As for long-term oncological outcomes and quality of life of taTME, there is a lack of high-level evidence. The encouraging result from TaLaR showed a declining trend in the rate of positive resection margins after taTME. Before further achievements from the international multicenter RCT COLOR III, ETAP-GRECCAR 11, and TaLaR, the priority of taTME clinical research is to guarantee the quality of radical resection and to ensure the safety of taTME, especially under the background that COLOR III has changed its primary outcome from CRM to three-year LR.

## Author Contributions

All authors listed have made a substantial, direct, and intellectual contribution to the work and approved it for publication.

## Funding

This study was supported by the Shanghai Science and Technology Commission, 18ZR1424300 (RZ); the Shanghai Hospital Development Center, SHDC2020CR1026B (RZ); the Shanghai Health Commission, 2019SY058 (RZ); the National Natural Science Foundation of China, 82002475 (XC); and the Shanghai Sailing Program, 20YF1427700 (XC).

## Conflict of Interest

The authors declare that the research was conducted in the absence of any commercial or financial relationships that could be construed as a potential conflict of interest.

## Publisher’s Note

All claims expressed in this article are solely those of the authors and do not necessarily represent those of their affiliated organizations, or those of the publisher, the editors and the reviewers. Any product that may be evaluated in this article, or claim that may be made by its manufacturer, is not guaranteed or endorsed by the publisher.
